# Determinants of suboptimal hepatitis B vaccine uptake among men in the Republic of Korea: where should our efforts be focused: results from cross-sectional study

**DOI:** 10.1186/1471-2334-13-218

**Published:** 2013-05-14

**Authors:** Boyoung Park, Kui Son Choi, Hoo-Yeon Lee, Min-Son Kwak, Jae Kwan Jun, Eun-Cheol Park

**Affiliations:** 1 National Cancer Control Institute, National Cancer Centre, 323 Ilsan-ro, Ilsandong-gu, Goyang-si, Gyeonggi-do 410-769, Korea; 2 Department of Social Medicine, College of Medicine, Dankook University, Cheonan, Korea; 3 Graduate School of Public Health, Seoul National University, Seoul, Korea; 4 Department of Preventive Medicine & Institute of Health Services Research, Yonsei University College of Medicine, Seoul, Korea

**Keywords:** Hepatitis B vaccine, Male, Sociodemographics

## Abstract

**Background:**

Liver cancer is the second most-frequent cause of cancer death in Korea. Hepatitis B virus (HBV) infection is a major cause of liver cancer, and this disease is effectively prevented by HBV vaccination. This study was conducted to investigate factors associated with the lack of HBV vaccine uptake in the general adult male population in Korea.

**Methods:**

Data of men who participated in a nationwide cross-sectional interview survey were analyzed. A total of 2,174 men 40 years of age and older were interviewed between 2006 and 2008. None of the participants was infected with HBV or was experiencing sequelae of an HBV infection.

**Results:**

Only half (50.4%) of the men received one or more dose of the three-dose series of HBV vaccinations, and 32.5% received all three doses. Compared with men who had completed the vaccination regimen, non-vaccinated men were more likely to lack supplemental medical insurance for cancer (odds ratio = 0.66, 95% confidence interval: 0.52–0.84), have lower incomes (*p*-trend = 0.010), and be less educated (*p*-trend = 0.021). Lower education was also more prevalent in the non-vaccinated group compared with the incompletely vaccinated group. Those who had completed the vaccination regimen were likely to be more educated than those in the incompletely vaccinated group (*p*-trend = 0.044). The most commonly cited reason for not obtaining the HBV vaccine was lack of knowledge regarding the need for the vaccination. The number of men who cited this reason decreased as a function of education.

**Conclusions:**

It is important to develop strategic interventions targeted at less-educated men to increase uptake of a complete three-dose series of HBV vaccinations as a primary approach to preventing liver cancer.

## Background

In 2009, liver cancer was the second most-frequent cause of cancer death in the Republic of Korea (33.0/100,000 men and 11.4/100,000 women). In the same year, 47.9/100,000 men and 16.2/100,000 women developed liver cancer, accounting for 12.0% of cancer incidence in men and 4.3% in women. Although the 5-year survival rate for liver cancer has been increasing, it remains low compared with other cancer types [1]. Chronic hepatitis B virus (HBV) infection is a major risk factor for development of liver cancer [2]. Of the 6,027 cases of hepatocellular carcinoma (HCC), 4,856 were attributed to HBV infection, accounting for 68.1% of HCC cases and deaths in Korea [3]. Although its prevalence has decreased from 8.6% in 1980 [4] to 3.2% in 2009 [5], the Republic of Korea is a HBV endemic area.

Worldwide, the rate of liver cancer development due to chronic HBV infection was 0.1%/person-year in 1997 [6]. In Korea, the rate was 0.8%/year, and the 5-year cumulative incidence was 3% in 2009 [4]. Another study conducted in Korea reported that during the 20-year period from 1972 to 1992, 26% of HBV-infected individuals developed primary liver cancer [7].

The HBV vaccine was the first cancer-preventive vaccine, which prevented liver cancer [2]. To reduce the disease burden of HBV infection, a catch-up vaccination program for adults was conducted in Korea. The first catch-up program, implemented in 1985, targeted government employees, teachers, and their dependents. It was followed by the mass HBV vaccination campaign, conducted by the Korean government in 1988, targeting school-aged children and adolescents. In 1995, the nationwide HBV vaccination program for infants, using a 0, 1, and 6 month vaccine schedule, was implemented by the National Immunization Program as part of the Communicable Diseases Prevention Act. However, a catch-up vaccination program for adults has not been conducted since the first in 1985. Although vertical transmission of HBV is the main source of infection [8], horizontal transmission is also important in HBV-endemic areas [9]. In Korea, HBV transmission occurs commonly in adulthood, which could be prevented by HBV vaccination. This includes the spread of infection through sexual contact, non-sexual transmission among family members in the same household, direct contact during adulthood, and occupational/health-care related sources [8,10,11]. Therefore, catch-up vaccination of adults is critical for the control of HBV infection and subsequent liver cancer [8,10].

In 2009, in Korea, the prevalence of hepatitis B surface antigen (HBsAg) and its sequelae was much higher among adult male compared with adult women or teenagers [4], who had been chosen as the target group in the nationwide HBV vaccination program [12]. This suggests that the adult male population in Korea is at high risk for HBV infection and subsequent sequelae. Studies of the determinants of HBV vaccination in the general adult population are rare and focus on high-risk groups [13,14]. Therefore, this study was conducted to investigate factors associated with HBV vaccine uptake in the general adult male population in Korea.

## Methods

### Study population and data

The Korean National Cancer Screening Survey (KNCSS) is an annual nationwide population-based cross-sectional interview survey using multi-stage random sampling to investigate participation rates in screening for gastric, liver, colorectal, breast, and cervical cancers in representative samples. Based on target populations identified in an organized cancer screening program conducted by the Republic of Korea, cancer-free men 40 years and older and cancer-free women 30 years and older comprise the KNCSS-eligible population. Data on total number of participants, response rate, and general characteristics of the participants were discussed by Lee, *et al*. [10]. Informed consent was obtained from all participants. The present study analyzed the data of men who participated in the KNCSS between 2006 and 2008. Among the 2,441 men included initially, we excluded 152 subjects who, by self-report, had existing health problems related to hepatitis infection, (*i*.*e*., liver cirrhosis), were HBsAg-positive, and/or had chronic infection associated with hepatitis C. We further excluded 52 men due to the presence of hepatitis B surface antibodies (anti-HBs) prior to vaccination as well as 63 others due to missing information pertaining to major variables. Data from 2,174 men were used in the final analyses.

Vaccination status was classified into three categories according to self-reported information on the number of doses of HBV vaccine received. Complete vaccination was defined as receiving the three-dose series of HBV vaccines, incomplete vaccination was defined as receiving either one or two doses, and non-vaccinated was defined as having never been vaccinated against HBV. Sociodemographic characteristics, including age, area of residence, supplemental medical insurance for cancer, monthly income, highest level of education, self-reported health status, occupation, and marital status were considered as independent variables. Monthly individual income was classified into four categories: ≤$999, $1,000–$1,999, $2,000–$3,499, and ≥$3,500 (US currency). Non-vaccinated men were asked to choose one of the following reasons for not being vaccinated: “Did not know I need HBV vaccination”, “Put it off due to feeling annoyed about it”, “Forgot”, “Lack of time”, “Did not know the severity of the sequelae of HBV infection”, and “Other”.

### Statistical analysis

Polychotomous logistic regression was used to identify factors associated with complete and incomplete vaccination [15], and logistic regression was used to identify the factors that differentiated between the complete and the incomplete-vaccination groups. Interaction tests were also conducted by including interaction terms between independent variables. The distribution of the reasons for being non-vaccinated was analyzed as a function of highest level of education achieved. All statistical analyses were performed using the SAS software version 9.1 (SAS Inc., Cary, North Carolina, USA). This study was approved by the Institutional Review Board of the National Cancer Center, Korea.

## Results

A total of 2,174 men aged 40 years and older who participated in the KNCSS between 2006 and 2008 were included in our analysis. Sociodemographic characteristics are shown in Table 1. About 73% were in their 40s and 50s, and 63.3% had supplemental medical insurance for cancer. About 15% reported a monthly individual income of $999 or less, and 30.4% had 11 or fewer years of education (Table 1).

**Table 1:** Baseline sociodemographic characteristics of 2,174 participants, Korean National Cancer Screening Survey, 2006–2008

**Variables**	***n *(%)**	
Age (years)		
40-49	962	(44.3)
50-59	621	(28.6)
60-69	467	(21.5)
70-80	124	(5.7)
Area of residence^*^		
Metropolitan	1,025	(47.2)
Urban	905	(41.6)
Rural	244	(11.2)
Supplemental medical insurance for cancer		
Yes	1,375	(63.3)
No	788	(36.3)
Don't know	11	(0.5)
Monthly individual income (USD)		
≤ 999	324	(14.9)
1,000-1,999	614	(28.2)
2,000-3,499	899	(41.4)
≥ 3,500	337	(15.5)
Level of education (years)		
≤ 11	660	(30.4)
12-15	1,038	(47.8)
≥ 16	476	(21.9)
Self-reported health status		
Good	1,437	(66.1)
Moderate	551	(25.3)
Poor	186	(8.6)
Occupation		
Managerial and professional	416	(19.1)
Service and sales	830	(38.2)
Routine and manual	656	(30.2)
Long-term unemployed	272	(12.5)
Marital status		
Married	2,030	(93.4)
Unmarried	144	(6.6)

^*^Metropolitan population ≥ 1.2 million; Urban population ≥ 50,000; Rural population < 50,000.

**Figure 1:**
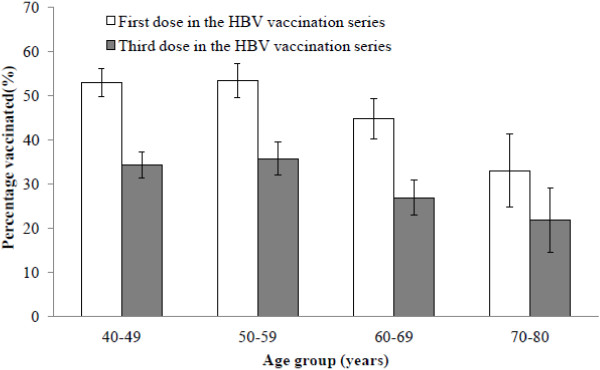
**Rate of vaccination series completion of hepatitis B virus (HBV) according to age among 2,174 men, Korean National Cancer Screening Survey, 2006–2008.** The complete vaccination rate was 32.5%, and 50.4% received one dose of the HBV vaccine.

Among the men who participated in the study, 7.5% received one dose of HBV vaccine, 10.4% received two doses, and 32.5% received all three doses (data not shown). In other words, approximately 64% of the 1,093 men who began the vaccination series completed it. The rate at which participants completed the vaccination series decreased as a function of age; 34.4% of the men in their 40s completed the series, while only 21.8% of men between the ages of 70 and 80 did so (Figure 1).

The relationship between HBV vaccine uptake and sociodemographic factors is presented in Table 2. The higher level of education was correlated with both the complete and incomplete HBV vaccination when compared with the non-vaccinated group (*p*-trend = 0.021 and < 0.001, respectively). Lower monthly individual income was also associated with a lower rate of complete vaccination (*p*-trend = 0.010) compared to that in unvaccinated men. Men who did not have supplemental medical insurance for cancer were less likely to be completely vaccinated compared with those who were not vaccinated at all (odds ratio = 0.66, 95% confidence interval: 0.52–0.84) (Table 2).

**Table 2:** **Factors associated with being non-vaccinated *versus *completely**^**† **^and incompletely^* ^**vaccinated against hepatitis B virus**^**‡ **^**among 2,174 men, Korean National Cancer Screening Survey, 2006–2008**

	**Complete vaccination**			**Incomplete vaccination**		
	**OR**	**(95% CI)**	***p*-trend**	**OR**	**(95% CI)**	***p*-trend**
Age (years)						
40-49	1.00	(referent)	0.322	1.00	(referent)	0.334
50-59	1.19	(0.93–1.52)		1.19	(0.88-1.61)	
60-69	0.88	(0.63–1.23)		1.42	(0.95-2.12)	
70-80	0.64	(0.37–1.11)		0.87	(0.43-1.77)	
Area of residence						
Metropolitan	1.00	(referent)	0.228	1.00	(referent)	0.686
Urban	1.05	(0.85–1.30)		0.87	(0.67-1.12)	
Rural	1.26	(0.90–1.76)		1.29	(0.87-1.92)	
Supplemental medical insurance for cancer						
Yes	**1.00**	**(referent)**		1.00	(referent)	
No	**0.66**	**(0.52–0.84)**		0.78	(0.59-1.05)	
Monthly individual income (USD)						
≤999	**0.62**	**(0.41–0.92)**	**0.010**	0.60	(0.35-1.01)	0.150
1,000–1,999	0.72	(0.51–1.01)		1.02	(0.67-1.53)	
2,000–3,499	0.78	(0.57–1.06)		1.00	(0.68-1.46)	
≥3,500	**1.00**	**(referent)**		1.00	(referent)	
Duration of education (years)						
≤11	**0.66**	**(0.47–0.93)**	**0.021**	**0.43**	**(0.28-0.65)**	**<0.001**
12–15	**0.70**	**(0.53–0.92)**		**0.62**	**(0.45-0.85)**	
≥16	**1.00**	**(referent)**		**1.00**	**(referent)**	
Self–reported health status						
Good	1.00	(referent)	0.877	1.00	(referent)	0.290
Moderate	1.11	(0.89–1.40)		0.96	(0.73-1.28)	
Poor	0.83	(0.56–1.22)		1.41	(0.93-2.15)	
Occupation						
Managerial and professional	1.00	(referent)		1.00	(referent)	
Service and sales	0.84	(0.63–1.11)		0.78	(0.56-1.10)	
Routine and manual	1.01	(0.73–1.41)		0.94	(0.64-1.40)	
Long–term unemployed	1.13	(0.73–1.75)		0.90	(0.52-1.55)	
Marital status						
Married	1.00	(referent)		1.00	(referent)	
Unmarried	0.74	(0.47–1.16)		0.57	(0.31-1.07)	

^*^Incomplete vaccination: received one or two doses of HBV vaccine.

^†^Complete vaccination: received the three-dosage HBV series vaccine.

^‡^Adjusted for list variables, type of health insurance, number of family members, smoking and drinking status and survey year.

OR: Odds ratio, CI: Confidence interval.

Duration of education was related to complete vaccination compared with incomplete vaccination; as education level decreased, the rate of complete vaccination decreased significantly (*p*-trend = 0.044, Table 3). Additionally, men between 60 and 69 years of age were more likely to receive the full three-dose regimen than were those 40–49 years of age and those who felt their health status was poor completely vaccinated more (Table 3).

**Table 3:** Factors associated with complete^* ^**compared with incomplete**^**† **^**vaccination**^**‡ **^**status among 2,174 men, Korean National Cancer Screening Survey, 2006–2008**

	**Incomplete vaccination**^**† **^***vs*.**		
	Complete vaccination^*^		
	**OR**	**(95% CI)**	***p*-trend**
Age (years)			
40-49	1.00	(referent)	0.128
50-59	1.00	(0.73–1.37)	
60-69	**1.60**	**(1.03–2.48)**	
70-80	1.27	(0.57–2.81)	
Area of residence			
Metropolitan	1.00	(referent)	0.561
Urban	0.83	(0.63–1.09)	
Rural	1.01	(0.66–1.55)	
Supplemental medical insurance for cancer			
Yes	1.00	(referent)	
No	1.19	(0.86–1.64)	
Monthly individual income (USD)			
≤999	1.01	(0.58–1.76)	0.567
1,000–1,999	1.45	(0.93–2.24)	
2,000–3,499	1.32	(0.89–1.96)	
≥3,500	1.00	(referent)	
Duration of education (years)			
≤11	**0.63**	**(0.40–0.98)**	**0.044**
12-15	**0.86**	**(0.62–1.21)**	
≥16	**1.00**	**(referent)**	
Self-reported health status			
Good	1.00	(referent)	0.311
Moderate	0.86	(0.63–1.16)	
Poor	**1.68**	**(1.04–2.71)**	
Occupation			
Managerial and professional	1.00	(referent)	
Service and sales	0.93	(0.65–1.32)	
Routine and manual	0.90	(0.60–1.37)	
Long-term unemployed	0.81	(0.46–1.45)	
Marital status			
Married	1.00	(referent)	
Unmarried	0.75	(0.38–1.50)	

^*^Incomplete vaccination: received one or two doses of HBV vaccine.

^†^Complete vaccination: received the three-dose HBV series vaccine.

^‡^Adjusted for list variables, type of health insurance, number of family members, smoking and drinking status and survey year.

OR: odds ratio, CI: confidence interval.

Table 4 shows the relationship between reasons for being non-vaccinated and the level of education. The most common reason for being non-vaccinated was no knowledge of the need for HBV vaccination, (cited by 40.3% of non-vaccinated men), followed by “Put it off due to feeling annoyed about it,” (19.8%), “Lack of time,” (18.0%), “Forgot,” (10.2%), and “Did not know the severity of HBV infection” (6.7%). Nearly half (46.5%) of those men with up to 11 years of education responded no knowledge of the need for HBV vaccination as the reason for being non-vaccinated. This decreased to 32.6% among men who had complete 16 or more years of education. Finally, the proportion that responded “Lack of time” increased nearly two-fold among men with 16 or more years of education compared with those who had 11 or fewer years of education, from 10.4% to 22.1% (Table 4).

**Table 4:** Reasons for not being vaccinated against hepatitis B virus (HBV) according to the level of education among 1,081 unvaccinated men, Korean National Cancer Screening Survey, 2006–2008

**Reasons**	**Total (%) (*n *= 1,081)**	**Duration of education (years)**		
		**≤11(%)**	**12-15(%)**	**≥16(%)**
		**(*n *= 385)**	**(*n *= 515)**	**(*n *= 181)**
Did not know I need HBV vaccination	40.3	46.5	38.5	32.6
Put it off due to feeling annoyed about it	19.8	17.4	21.4	20.4
Forgot	10.2	9.9	8.5	15.5
Lack of time	18.0	10.4	22.3	22.1
Did not know the severity of the sequelae of HBV infection	6.7	9.4	4.9	6.1
Other	5.0	6.5	4.5	3.3

## Discussion

This is the first study to investigate the factors associated with HBV vaccine uptake, an effective medical intervention for the prevention of liver cancer in the general adult male population in Korea. In the current study, factors associated with complete HBV vaccination included higher income, higher level of education, and supplemental medical insurance for cancer. Level of education was significantly associated with incomplete *versus* non-vaccinated status with complete *versus* incomplete vaccination status.

The three-dosage series of HBV vaccine is highly recommended for maximum effectiveness. Indeed, after receiving the complete three-dosage series of HBV vaccination, seroprotection rates approached 100% among healthy children and 95% among healthy adults [16]. In Korea, HBV vaccination is offered by a wide range of healthcare facilities, including local public health clinics, primary healthcare settings, and tertiary hospital facilities and the costs of HBV vaccination for adults are relatively low (3–30 $US). HBV vaccine costs are considerably lower when administered in public health clinics than in private hospitals. However, half of the men in the present study had never been vaccinated against HBV, and only 32.5% of the participants received the complete three-dose vaccination, despite the relatively high incidence of HBV infection, liver cirrhosis, and liver cancer among men in Korea. It is noteworthy that 39.8% of Korean women received the complete vaccination, a higher rate than among Korean men. One reason for this difference might be the fact that the program for the prevention of vertical HBV transmission targeted all pregnant women in Korea [17].

To address the secondary prevention of liver cancer, the National Cancer Screening Program in Korea began an initiative for liver cancer screening in 2003. The program provides liver cancer screening (alpha-fetoprotein and ultrasonography) every 6 months for persons aged 40 or older who are positive for HBsAg, anti-HCV, or liver cirrhosis [18]. Although the screening program was implemented for secondary prevention, the importance of the primary prevention of liver cancer through HBV vaccination has not been sufficiently emphasized or targeted at the adult population.

Level of education is strongly related to the amount of health information sought and understood, especially in the context of cancer-related issues. Indeed, educational level can influence health-communication behaviors [19] and the acquisition and integration of new information [20]. In this study, a higher level of education was positively associated with HBV vaccine uptake and it was also positively correlated with completing the three-dosage HBV series vaccination. Similarly, a study of immigrants to the United States showed that attainment of higher levels of education had a positive effect on HBV vaccination uptake [5]. Thus, it may be suggested that a higher level of education is important for increasing HBV vaccine uptake among immigrants and the general population, particularly in countries that provide national health insurance coverage, including Korea.

Completion of the three-dosage HBV vaccination series has been associated with adherence to medical recommendations. Education has been reported to influence physician–patient interaction, and patients with higher education levels tend to be more likely to adhere to medical treatment, thereby leading to better health outcomes [21]. Higher income and having supplemental medical insurance for cancer have been shown to increase both accessibility to health care services and the quality of care [22]. HBV infection in adulthood is common in high-prevalence areas such as Korea [9], and those with low socioeconomic status have a higher incidence of HBV infection [23]. Taking these factors into consideration, the low rates of HBV vaccination among people who are less educated, have lower incomes, and/or are not covered by supplemental insurance for cancer continue to be issues that need to be resolved.

Our study revealed that the most common reason for not being vaccinated was lack of fundamental knowledge regarding the necessity of HBV vaccination, which was cited by 40% of non-vaccinated participants. Similarly, a previous study found that lack of awareness about vaccination recommendations was the main barrier to becoming vaccinated [24]. Moreover, as the level of education decreased, the proportion of men answering “Did not know I need HBV vaccination” increased. This finding may reflect disparities in how healthcare information is communicated. People with lower education levels tend to be less aware of their hepatitis-infection status when compared with more educated people [25]. This in turn could affect liver cancer screening, because only high-risk groups, such as hepatitis carriers or liver cirrhosis patients, are targeted by the National Cancer Screening Program in Korea. As a result, it is not surprising that the burden of liver disease is much greater among less educated people. In Korea, inequality in liver cancer mortality exists; higher mortality rates due to liver cancer have been associated with lower levels of education [26]. Our results indicate the importance of focus efforts on health education of less educated groups and underscore the necessity of increasing HBV vaccination uptake.

This study has several potential limitations. HBV infection status, which was used as an exclusion criterion, was based on self-reports of previous HBV infection instead of the results of serology tests. Therefore, we did not confirm whether participants had been exposed to previous acute or chronic infection with HBV (HBsAg and anti-HBc), introducing the possibility of erroneous data resulting from information bias. Additionally, data on vaccination history and sociodemographic characteristics were based solely on self-reporting. Measurement of serum total anti-HBs may be a more reliable means of determining the vaccination status of the participants. However, considering 10-15% of those who complete three-dosage series of HBV vaccine fail to response (anti-Hbs level below 10 mIU/ml) [27], it also has some limitations. We did not review participants’ medical records and consequently could not verify HBV vaccination status. Previous studies have reported different results regarding the reliability of self-reported vaccination history. Some suggested that self-reported vaccination history and personal information are reliable in that they are consistent with the health information documented in medical records [28,29] but a study have found opposite results [30]. Information bias may have led to inaccurate information about vaccination status. Furthermore, despite the fact that both recombinant and plasma-derived HBV vaccines were used in Korea until 2004, we did not ask participants about the type of HBV vaccine received. The type of vaccine administered determines which one of two standard schedules is used; the 0, 1, 6 month schedule is applied when vaccinating with recombinant HBV, whereas the 0, 1, 2 month schedule is applied when using plasma-derived vaccine. Such differences between the two HBV vaccines may have affected the accuracy of the reports of vaccination history.

Despite these limitations, this study has several strengths. First, by identifying the predictors of incomplete and complete HBV vaccination, our results will be useful in developing strategies to increasing HBV vaccination uptake in the general adult male population. Second, the results are representative of the Korean male 40 years of age or older due to our use of a population-based sampling approach.

HBV vaccination is an important intervention used to decrease the incidence of liver cancer, and universal HBV vaccination is conducted in many Asian countries [21]. According to our results, the rate of HBV vaccination among Korean men aged 40 years or older was low between 2006 and 2008. HBV vaccination uptake was related to level of education, supplemental medical insurance for cancer, and monthly income; findings showed that educational level was associated with complete vaccine uptake among men who had started the HBV vaccination series. Lack of knowledge of the necessity of HBV vaccination was the most common reason for non-vaccinated status, and data showed that this was influenced by participants’ educational level. Although only 1–5% of the adult population are chronically infected following an acute infection [27], common means of horizontal transmission (*i*.*e*., sexual contact, non-sexual transmission, occupational/health-care related routes) are very common in developing and endemic countries [6]. Therefore, adults should also be encouraged to receive HBV vaccination to prevent HBV transmission by development of herd immunity. Although the Korean government has conducted HBV vaccination programs for primary prevention of liver cancer, certain classes of adults are neglected because the focus is restricted to children. Thus, additional health promotion and education strategies are needed to enhance HBV vaccine uptake; these strategies should target specifically men with a lower level of education.

## Conclusions

Hepatitis B virus (HBV) infection and its sequelae are major global health problems. Although vertical transmission of HBV is the main source of infection, horizontal transmission is also important in HBV-endemic areas, especially in Korea, where HBV infection commonly occurs during adulthood. To increase the rate of complete HBV vaccination it may be necessary to develop tailored approaches that take into account the various factors associated with vaccine uptake. In the male population, the level of education has been consistently associated with complete vaccination compared with non- and incomplete vaccination status. A lack of awareness about the necessity of HBV vaccination was the most common reason for being non-vaccinated; the proportion of men citing this reason decreased as a function of education. Strategies aimed at increasing knowledge of the need for HBV vaccination and increasing complete vaccination rates should be developed and targeted at Korea’s less educated male population, as vaccination is the primary preventative intervention for liver cancer.

## Abbreviations

HBsAg: Hepatitis B surface antigen; HBV: Hepatitis B virus; KNCSS: Korean national cancer screening survey.

## Competing interests

The authors declare that they have no competing interests.

## Authors’ contributions

PB has been involved in analyzing the data and drafting the manuscript. CKS and LHY have made contributions to conception and design of the study. KMS has made contributions to acquisition of data and analysis. JKJ has given final approval of the version to be published. PEC has made contributions to acquisition of data. All authors read and approved the final manuscript.

## Pre-publication history

The pre-publication history for this paper can be accessed here:

http://www.biomedcentral.com/1471-2334/13/218/prepub
